# Evaluation of adherence to national regulations for documentation of limitations of life-sustaining treatments after a limited intervention at a Swedish hospital

**DOI:** 10.1016/j.resplu.2026.101492

**Published:** 2026-09-17

**Authors:** Eva Piscator, Charlotte Ekestam, Lena Bremer, Umut Heilborn

**Affiliations:** a Department of Medicine Solna, Karolinska Institutet, Sweden; b Department of Emergency Medicine, Capio St Göran’s Hospital, Stockholm, Sweden; c Department of Medicine, Capio St Göran’s Hospital, Stockholm, Sweden

**Keywords:** DNACPR, DNAR, Do not attempt cardiopulmonary resuscitation, Legislation and jurisprudence, Resuscitation orders, Withholding treatment

## Abstract

**Aim:**

The aim of this study was to improve adherence to the Swedish National Board of Health and Welfare’s regulations for documenting decisions regarding Limitations of Life-Sustaining Treatments at Capio St. Göran’s Hospital in Stockholm following a limited intervention.

**Methods:**

This was a before-and-after-study. The intervention took place in June 2023 and consisted of three parts: 1. Implementation of a modified digital medical record form for documentation of decisions regarding limitations of Life-Sustaining Treatments. 2. A brief educational intervention for physicians at the hospital. 3. Publication of an updated hospital protocol for decisions on limitations of Life-Sustaining Treatments. We analysed 100 randomly selected documentations of limitation of Life-Sustaining Treatments in adult patients before the intervention, and 98 after the intervention. Adherence to individual mandatory criteria and overall adherence to the regulatory requirements was evaluated.

**Results:**

Overall adherence to all documentation regulations increased from 1% to 63% (*p* < 0.01). Grounds for the decision were well documented both pre- and post-intervention (99% and 97%, *p* > 0.99). All other individual criteria improved: consultation with another licenced caregiver (65–89%, *p* < 0.01), dialogue with the patient or motivation why not (24–89%, *p* < 0.01), information to the family or motivation why not (23–74%, *p* < 0.01), the view of the patient (14–86%, *p* < 0.01) and the view of the family (4–70%, *p* < 0.01).

**Conclusion:**

A moderate intervention in terms of cost and work effort for the organisation can significantly improve adherence to regulations regarding documentation of limitations of Life-Sustaining Treatments, although further improvement is needed.

## Introduction

The legislation and clinical practises regarding decisions for limitations of Life-Sustaining Treatments (LST) vary greatly worldwide.^1^, ^2^, ^3^ Previous publications have shown variable, but generally low, adherence to both ethical principles emphasising patient autonomy with shared decision making, as well as low adherence to regulatory requirements in several countries, including Sweden.^4^, ^5^, ^6^, ^7^, ^8^, ^9^, ^10^, ^11^, ^12^

The Swedish regulations of the National Board of Health and Welfare^13^ require that the physician responsible for the limitation of LST decision, documents the following mandatory headings:

1. The grounds for the decision, 2. Consultation with another licenced healthcare professional, 3. Dialogue with the patient, or a motivation as to why a dialogue has not taken place, 4. Communication with the patient’s family, or a motivation as to why a dialogue has not taken place, 5. The view of the patient and 6. The view of the family in the matter. Documentation of a motivation as to why communication with the patient́s family did not take place is not mandatory by regulations but was considered significant and therefore added as mandatory to the modified medical record form.

In 2019, a pilot study was conducted at Capio St. Göran’s Hospital, Stockholm, Sweden showing poor adherence to the regulations (not published). The aim of this study, therefore, was to improve adherence to regulations for the documentation of limitation of LST decisions at this hospital, by implementing a modified medical record form, conducting a brief educational intervention, and updating the hospital protocol for decisions on limitation of LST.

## Methods

### Study design, population and data collection

This before-and-after study included adults with documentation of limitation of LST decisions at Capio St. Göran’s Hospital, Stockholm, Sweden. The hospital in central Stockholm is a general and emergency hospital with a broad spectrum of medical specialties providing both acute and elective care with public funding. It has a catchment area of approximately 420,000 inhabitants and had about 90,000 visits to the emergency department in 2025. It holds medical wards for Emergency medicine, Internal medicine, Neurology and Stroke, Cardiology, Oncology, Surgery, Urology, Orthopaedics, Anaesthesia-and Intensive care and Obstetrics and Neonatology.

Sample size calculation was based on a pilot study performed in 2019 with analysis of 50 retrospectively and randomly selected adult decisions regarding limitation of LST. Results showed variable but overall low adherence to the regulations. The power calculation was based on the assumption that the intervention would increase adherence to regulations by 20 percentage points. The study population was identified using the hospital’s IT systems (Insight and Power BI) by extracting all medical record forms for documentation of limitation of LST decisions. Inclusion criteria were patients ≥18 years receiving inpatient care in 2022 (pre-intervention), and in the last quarter (Q4) of 2023 (post-intervention). Information regarding age, sex, and the medical clinic responsible for the decision was retracted by the hospital’s quality controller based on the IT systems. Since analysis of the forms required manual interpretation, it was not feasible to include all limitation of LST decisions that were made. We therefore randomly selected and analysed 100 documentations pre-, and 100 post-intervention for analysis of adherence to the legislation in the documentation.

### Intervention

The intervention consisted of three parts, all carried through during June 2023: modification of the digital medical record form for documentation of decisions regarding limitation of LST, a brief educational intervention for physicians at the hospital and, finally, the publication of an updated hospital protocol for decisions on limitation of LST.

The digital forms used before and after intervention are shown in Fig. 1, Fig. 2. The modified digital medical record form was designed to more clearly indicate which documentation is mandated by regulations. The headings of the form were based on the legal requirements. The main change was that the form could not be signed unless all mandatory fields we filled. Some headings had predefined selections combined with free-text areas, whereas others were free-text only. The form could not be signed unless all mandatory headings were completed. Mandatory free-text headings required the entry of at least one character. The modified medical form adapted to the physician’s input. For instance, when filling the field “has information been provided to the family”: checking “yes” opens a new field “to whom and when” while checking “no” opens the field “reason for not informing family”. The subsequent adaptive questions are mandatory according to regulations. The modified form also has the option “no limitation of LST”. This field is new and in accordance with the regulations that dictate that LST decisions must be reconsidered at every hospital admission and previous limitations can be revoked. The pre-intervention form contained only a few of the headings mandated by regulations, and the headings did not clearly indicate which documentation is required by law. The only previous mandatory heading was the name of the physician who documented the decision, date and time for the documentation.

**Fig. 1:**
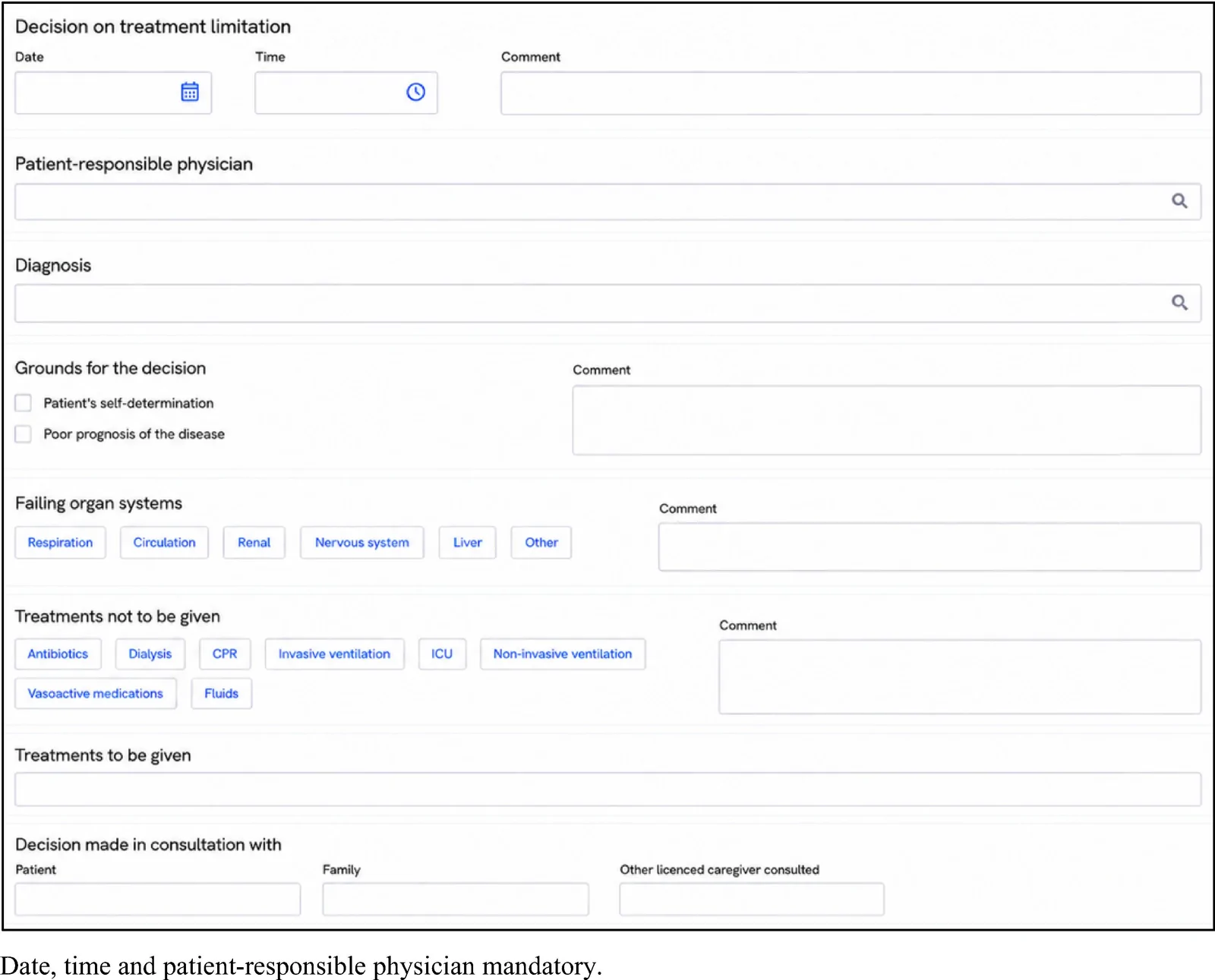
**The pre-intervention medical record form for Limitations of Life-Sustaining Treatments at Capio St. Göran’s Hospital in Stockholm**. Date, time and patient-responsible physician mandatory.

**Fig. 2:**
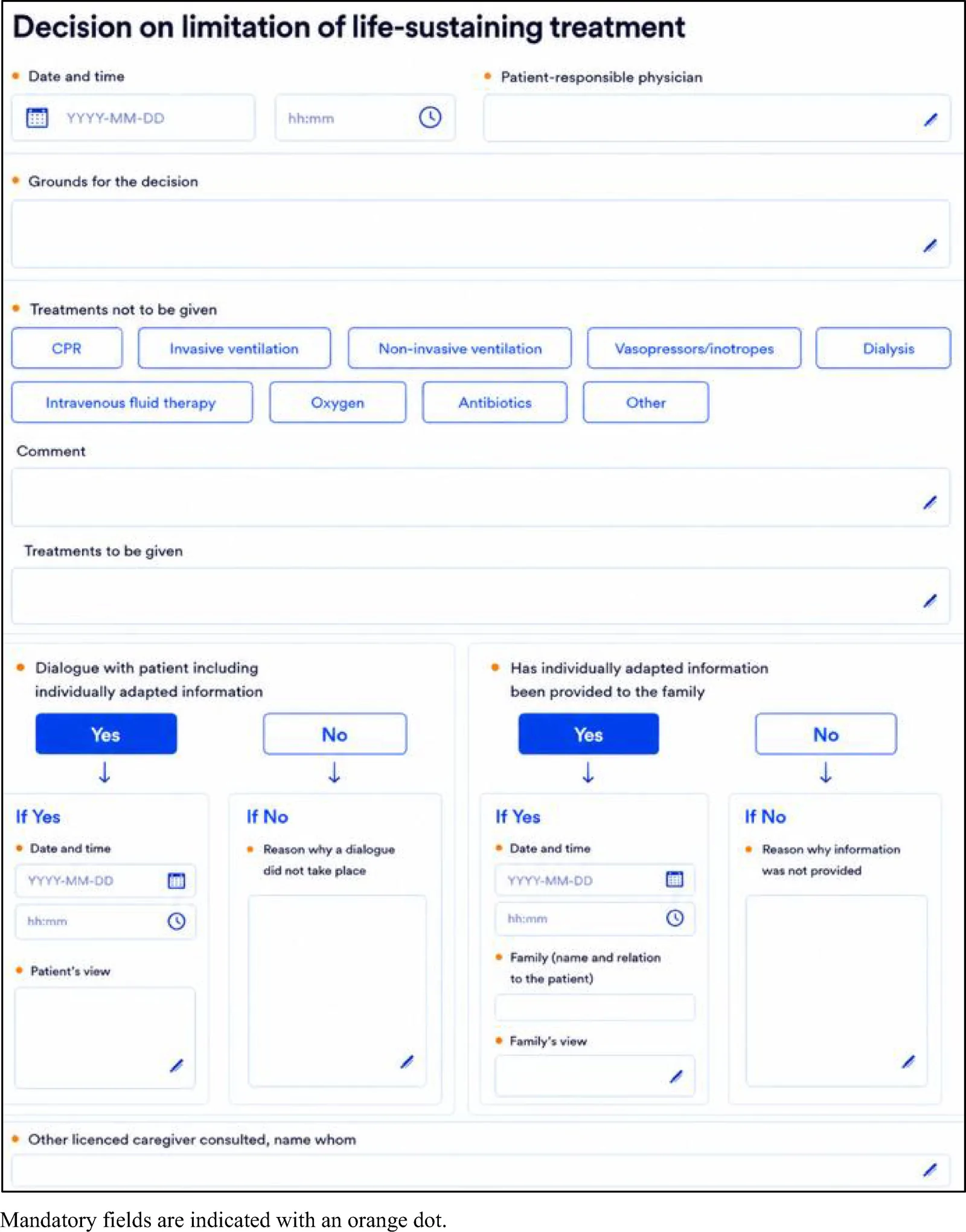
**The post-intervention medical record form for Limitations of Life-Sustaining Treatments at Capio St. Göran’s Hospital in Stockholm**. Mandatory fields are indicated with an orange dot. (For interpretation of the references to colour in this figure legend, the reader is referred to the web version of this article.)

The educational intervention consisted of a lecture (20–30 min) with physicians at staff meetings for each medical department separately. The lecture informed on the national regulations, the results of the pilot study, the modified medical record form and the updated hospital protocol. Attendance to the lectures was not monitored.

The updated hospital protocol for decisions on limitation of LST contained clinical practise, information about legislation and regulations for documentation, as opposed to the former protocol which focused on clinical practise only.

### Data analysis

Each medical record form was assessed for adherence to individual mandatory criteria and to overall adherence to regulations according to:

1. Grounds for the decision:
  Pre-intervention: documentation in free text or selected from the predefined list.
  Post-intervention: documentation in free text.
2. Consultation with another licenced caregiver:
  Documentation in free text.
3. Dialogue with the patient:
  Documentation of dialogue with the patient, or an explanation as to why no dialogue with the patient has taken place.
4. Communication with the family:
  Documentation of given information, or an explanation as to why no information was given to the family.
5. Documentation of the patient’s view on the matter, or an explanation as to why no dialogue with the patient has taken place.
6. Documentation of the family’s view on the matter, or an explanation as to why no information was given to the family.

The content of free-text entries was analysed pre-intervention as the medical record form did not have clear headings related to what was obliged by law, and the information could be written in free text. As long as responses were relevant to the content of the regulations, they were considered valid. Post-intervention, free-text analysis was restricted to those headings demanding free-text. As long as the response was relevant to the heading, it was considered valid. Cases of uncertainty was discussed with a senior researcher until consensus was reached.

### Statistical analysis

Descriptive statistics were reported as the median and interquartile range. Proportions were reported with 95% confidence intervals calculated using the Wilson score method. Binary variables were compared using Chi-squared, or Fisher’s exact test in case of 5 or less values per cell. Significance tests were two-sided with a significance level of 0.05. Random selection of patients was conducted with Microsoft Excel®. Analyses were performed using Stata 13 for Windows (Stata Corp, College Station, TX).

### Ethical approval

The study was approved by the Swedish Ethical Review Authority (2022-04985-01).

## Results

In 2022, 4531 limitation of LST decisions, and in Q4 of 2023, 1317 limitation of LST decisions were documented at Capio St Göran’s hospital, see Table 1 for details. The most common limitation was Do-Not-Attempt-Cardiopulmonary-Resuscitation (DNACPR) followed by limitation to invasive ventilation. Around one third were documented in the Emergency department and the rest in clinical wards, with predominance at clinics within the cluster of Internal medicine. The randomly selected pre- and post-intervention cohorts were shown in Table 2. Distribution of variables such as age, sex, type of decision and clinic were similar to the total cohort, although no statistical method was used to show representativity 1. No exclusions were made in the pre-intervention cohort, there were two exclusions due to the decision “No treatment limitation” in the post-intervention cohort.

**Table 1:** Characteristics of all patients with a decision to limit life-sustaining treatments in 2022 and the last quarter of 2023 at Capio St. Göran’s Hospital, Stockholm, Sweden.

	**Pre-intervention** **Year 2022** ***n* = 4531**	**Post-intervention** **Last quarter of 2023** ***n* = 1317**
**Patient characteristics**		
Age, median [IQR]	86 [19;108]	86 [42;102]
Women, *n* (%)	2338 (52)	694 (53)

**Limitation of Life-Sustaining Treatment**	***n* (%)**	***n* (%)**
DNACPR	4396 (99)	1013 (99)
No referral to Intensive Care Unit	2499 (57)	–a
No invasive ventilation	2777 (63)	655 (64)
No non-invasive ventilation	769 (17)	196 (19)
No dialysis	1597 (36)	372 (36)
No treatment with vasopressors	863 (20)	268 (26)
No treatment with antibiotics	106 (2)	32 (3)
No treatment with intravenous fluids	80 (2)	30 (3)
No treatment with supplemental oxygen	0	16 (2)

**Location**	***n* (%)**	***n* (%)**
Emergency department	1740 (38)	477 (36)
– Medicine section^b^	1396 (31)	393 (30)
– Surgery section	261 (6)	70 (5)
– Orthopaedic section	83 (2)	14 (1)
Inpatient care	2757 (61)	835 (63)
– Emergency medicine clinic	644 (14)	174 (13)
– Internal Medicine clinic	935 (21)	253 (19)
– Neurology clinic	230 (5)	71 (5)
– Cardiac clinic	492 (11)	153 (12)
– Surgery and Oncology clinic	310 (7)	118 (9)
– Orthopaedic clinic	145 (3)	66 (5)
– Intensive Care Unit	1 (1)	0
Outpatient care	34 (0.8)	5 (0.4)

*Abbreviations:* DNACPR, Do-Not-Attempt-Cardiopulmonary-Resuscitation.

a Option removed in the postintervention medical record form since the hospital introduced a High Dependency Unit with the same medical treatment as the Intensive Care Unit, except for invasive ventilation and dialysis.

b With subsections Emergency medicine, Cardiology and Neurology.

**Table 2:** Characteristics of patients in the randomly selected pre-and postintervention cohorts at Capio St. Göran’s Hospital, Stockholm, Sweden.

	**Pre-intervention** **Year 2022** ***n* = 100**	**Post-intervention** **Last quarter of 2023** ***n* = 100**
**Patient characteristics**		
Age, median [IQR]	89 [51;103]	87 [42;100]
Women, *n* (%)	45 (45)	52 (52)

**Limitation of Life-Sustaining treatment**	**Total *n* = 100** ***n* (%)**	**Total *n* = 98**^a^ ***n* (%)**
DNACPR	99 (99)	96 (98)
No referral to Intensive Care Unit	54 (54)	–b
No invasive ventilation	55 (55)	61 (62)
No non-invasive ventilation	14 (14)	23 (23)
No dialysis	40 (40)	36 (37)
No treatment with vasopressors	22 (22)	26 (27)
No treatment with antibiotics	2 (2)	1 (1)
No treatment with intravenous fluids	2 (2)	1 (1)
No treatment with supplemental oxygen	0	0

**Location**	***n* (%)**	***n* (%)**
Emergency department	36 (36)	41 (41)
– Medicine section^c^	32 (32)	35 (35)
– Surgery section	3 (3)	5 (5)
– Orthopaedic section	1 (1)	1 (1)
Inpatient care	63 (63)	59 (59)
– Emergency medicine clinic	12 (12)	15 (15)
– Medicine clinic	19 (19)	16 (16)
– Neurology clinic	5 (5)	4 (4)
– Cardiac clinic	10 (10)	12 (12)
– Surgery and Oncology clinic	13 (13)	5 (5)
– Orthopaedic clinic	4 (4)	7 (7)
Outpatient care		0
– Endoscopy unit	1 (1)	

*Abbreviations:* DNACPR, Do-Not-Attempt-Cardiopulmonary-Resuscitation.

a Two documents were No treatment limitation.

b Option removed in the postintervention medical record form since the hospital introduced a High Dependency Unit with the same medical treatment as the Intensive Care Unit, except for invasive ventilation and dialysis.

c With subsections Emergency medicine, Cardiology and Neurology.

### Adherence to regulations for the documentation of limitation of LST decisions

Complete adherence to all mandatory documentation requirements improved significantly from 1% pre- to 63% post-intervention (*p* < 0.01, 95% CI 0.2–5.4% and 53.2–71.8% respectively), as illustrated in Fig. 3. Grounds for the decision was well documented both pre-and post-intervention (99% (95% CI 94.6–99.8%) and 97% (95% CI 91.5–99%) respectively, p-value > 0.99). Documentation of consultation with another licenced caregiver increased from 65% to 89% (*p* < 0.01, 95% CI 55.3–73.6% and 81.4–93.7% respectively). Regarding the documentation of a dialogue with the patient, and information to the family, a more than three-fold improvement was seen (24% (95% CI 16.7–33.2%) to 89% (95% CI 81.4–93.7%) and 23% (95% CI 15.8–32.2%) to74% (95% CI 64.6–81.6%) respectively, *p* < 0.01 for both). Starting from lower baseline values, even higher improvements was seen for documentation of the view of the patient and family (14% (95% CI 8.5–22%) to 86% (95% CI 77.9–91.5%) and 4% (95% CI 1.6–9.8%) to 70% (95% CI 60.4–78.1%) respectively, *p* < 0.01 for both). Adherence to subcomponents of the dialogue with the patient and communication with the family regulations can be seen in Fig. 4.

**Fig. 3:**
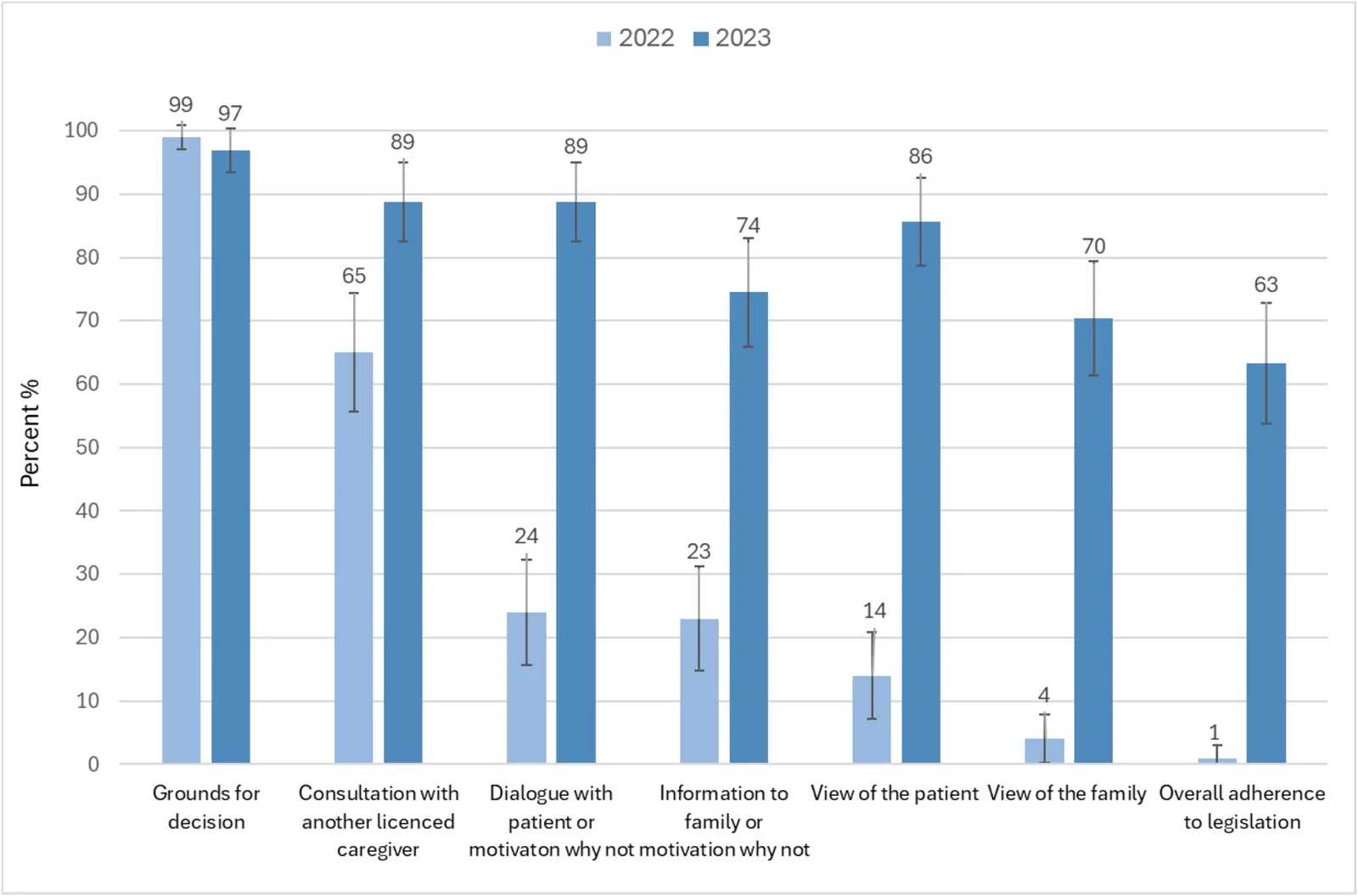
**Fulfilment of documentation requirements according to Swedish regulations before and after implementation of a new medical record form, conduction of a limited educational intervention, and publication of an updated hospital routine for decisions on Limitations of Life-Sustaining Treatments at Capio St Görans hospital, Stockholm, Sweden**. Range bars indicate 95% confidence intervals. *P*-values compare pre-intervention 2022 and post-intervention quartal 4 2023. All groups *p* < 0.01 except Grounds for decision, *p* > 0.99.

**Fig. 4:**
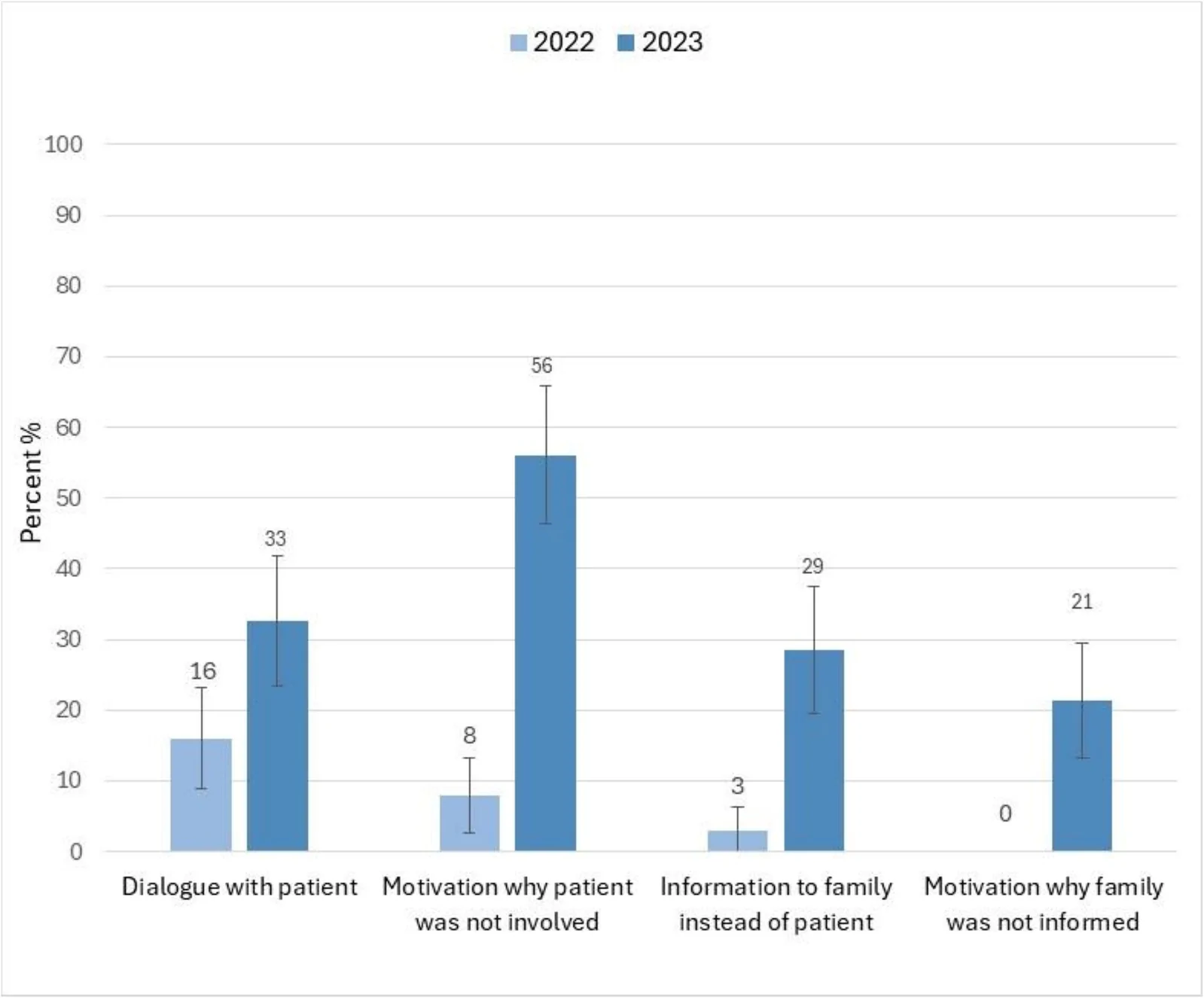
**Fulfilment of sub-components of regulations for adherence to subcomponents of the dialogue with the patient and communication with the family according to Swedish regulations before and after implementation of a new medical record form, conducting a limited educational intervention, and publication of an updated hospital routine for decisions on limitations of Life-Sustaining Treatments at Capio St Görans hospital, Stockholm, Sweden**. Range bars indicate 95% confidence intervals. *P*-values compare pre-intervention 2022 and post-intervention quartal 4 2023. All groups *p* < 0.01 except Dialogue with patient, *p* > 0.99.

## Discussion

This study has shown that adherence to the National Board of Health and Welfare’s regulations regarding documentation of decisions to forgo life-sustaining treatment significantly improved with an intervention consisting of implementation of a modified medical record form for the documentation of decisions regarding limitation of LST, a brief educational intervention for physicians at the hospital and publication of an updated hospital protocol for limitation of LST decisions.

A moderate work effort was required by the IT department to modify the previous digital form within the already existing system in accordance with our instructions. In addition, the process of filling a digital form regarding LST was already familiar to the physicians of our hospital. We therefore conclude that a moderate intervention in terms of cost and labour can significantly improve adherence to regulations regarding limitations of LST.

Complete adherence to all documentation regulations was surprisingly low before the intervention (1%), even lower than 10% shown in a previous study from a University hospital in the same region.^7^ In view of that, post-intervention levels of 63% could be considered a substantial achievement. However, much improvement remains before adherence to the regulatory requirements can be considered fulfilled. The practise of documenting the grounds for limitation of LST decisions appears to be well established, and was higher than the 89% shown in the previously mentioned study.^7^ Documentation of consultation with another licenced caregiver increased from moderate (65%) to seemingly acceptable (89%). Fulfilment of these two parameters is important for the transparency of the decision as to communicating the medical rationale on which the decision is based. They provide relevant information for re-assessing the decision should the patient’s medical condition change and constitute a basis for renewed dialogue with the patient and family. As previous studies have shown that decisions on limitation of LSTs are complex and prediction of outcome is perceived as difficult,^14^, ^15^, ^16^ clarifying the medical ground for the decision and consultation with a licenced colleague serve as control functions of the individual physicians' assessments.

Swedish legislation emphases the patient’s right to autonomy and shared decision making.^13^, ^17^, ^18^, ^19^ In this study, all parts involving documentation of a dialogue with the patient and family increased significantly to levels that could seem acceptable, at least as a starting point. If this increase was an effect of an increase in high-quality dialogues being conducted, and not just that the documentation of the dialogue increased, it would represent a considerable positive impact for patients and families. Subsequent studies may investigate this issue further.

Limitations of the study include that we assessed the effect based on a small sample rather than all documentations and we did not statistically analyse representativity.

A further limitation of the study is that we cannot separate the effects of the three different components of the intervention. Surveys among Swedish physicians have shown a lack of knowledge about the National Board’s regulations, as well as lack of knowledge of the journal templates in their medical record systems.^4^, ^6^ It is possible that our interventions meet the knowledge gap in these topics. However, previous studies have shown conflicting results on the effectiveness of interventions, including education, on limitation of LST dialogues and their documentation.^12^, ^20^, ^21^, ^22^ Factors indicating that the educational intervention in our study was unlikely to have a major effect on the results were that only physicians on duty were present at the lecture, and that it was not repeated on several occasions. Neither do we believe that the publication of an updated and more informative hospital protocol for decisions on limitation of LST had a major effect, since the experience of the authors is that only a limited number of physicians read the local protocolss.

A systematic review conducted by Field et al.,^22^ revealed that with interventions that aimed at enhancing the appropriate use and outcomes of DNACPR decisions, standardised documentation contributed to improving both the frequency and quality of DNACPR decision documentation. Another study by Haire et al.,^23^ evaluated a national intervention in Wales with the aim of enhancing documentation of DNACPR decisions. The intervention consisted of a new DNACPR form (in paper) and a more extensive educational intervention. The new DNACPR form had mandatory text boxes for documentation of the grounds for the decision and discussions with patients and significant others. The study showed improved written documentation, and the authors attributed the results to the combined effect of the new mandatory form and the educational intervention. Our interpretation of the results in our study is that the major contributing factor was the modified medical record form, which was designed to clearly indicate which documentation is mandated by regulations and impossible to sign digitally without filling all mandatory sections. The educational effort and publication of a more informative hospital protocol possibly provided additional support.

Another limitation of this study was that before the intervention, a dialogue with the patient and family could have been documented in the medical record outside of the form (especially paragraphs without separate headings), resulting in falsely high improvement in adherence to regulations. This risk however is considered low, as the hospital, since many years, clearly has formulated that a limitation of LST is only valid if documented in the specific medical form designed for that purpose.

We did not follow up whether the improvement in adherence lasted over time. While the educational intervention may fade over time, the modification of the digital form, with mandatory entries, remain and we therefore speculate that the major part of the improvement persists, although this has to be further studied.

We hypothesise that the nature of this study makes it generalisable to other health systems using electronic medical record systems, and preferably those that enable forms with mandatory headings.

## Conclusion

This study has shown that a limited intervention was associated with a significant improvement in adherence to the National Board of Health and Welfare’s regulations for the documentation of decisions to refrain life-sustaining treatments. Despite this, there is room for further improvement.

## Declaration of generative AI and AI-assisted technologies in the manuscript preparation process

During the preparation of this work the authors used Chat GPT in order to translate the Swedish medical LST forms into English. After using this tool, the authors reviewed and edited the content as needed and take full responsibility for the content of the published article.

## CRediT authorship contribution statement

**Eva Piscator:** Writing – original draft, Supervision, Methodology, Investigation, Formal analysis, Conceptualization. **Charlotte Ekestam:** Writing – review & editing, Formal analysis, Data curation, Conceptualization. **Lena Bremer:** Writing – review & editing, Formal analysis, Data curation, Conceptualization. **Umut Heilborn:** Writing – review & editing, Supervision, Formal analysis, Conceptualization.

## Declaration of competing interest

The authors declare that they have no known competing financial interests or personal relationships that could have appeared to influence the work reported in this paper.
